# Actual and undiagnosed HIV prevalence in a community sample of men who have sex with men in Auckland, New Zealand

**DOI:** 10.1186/1471-2458-12-92

**Published:** 2012-02-01

**Authors:** Peter JW Saxton, Nigel P Dickson, Richard Griffiths, Anthony J Hughes, John Rowden

**Affiliations:** 1 AIDS Epidemiology Group, Department of Preventive and Social Medicine, University of Otago Medical School, PO Box 913, Dunedin, New Zealand; 2 Research Unit, New Zealand AIDS Foundation, PO Box 6663, Wellesley Street, Auckland, New Zealand

## Abstract

**Background:**

The prevalence of HIV infection and how this varies between subgroups is a fundamental indicator of epidemic control. While there has been a rise in the number of HIV diagnoses among men who have sex with men (MSM) in New Zealand over the last decade, the actual prevalence of HIV and the proportion undiagnosed is not known. We measured these outcomes in a community sample of MSM in Auckland, New Zealand.

**Methods:**

The study was embedded in an established behavioural surveillance programme. MSM attending a gay community fair day, gay bars and sex-on-site venues during 1 week in February 2011 who agreed to complete a questionnaire were invited to provide an anonymous oral fluid specimen for analysis of HIV antibodies. From the 1304 eligible respondents (acceptance rate 48.5%), 1049 provided a matched specimen (provision rate 80.4%).

**Results:**

HIV prevalence was 6.5% (95% CI: 5.1-8.1). After adjusting for age, ethnicity and recruitment site, HIV positivity was significantly elevated among respondents who were aged 30-44 or 45 and over, were resident outside New Zealand, had 6-20 or more than 20 recent sexual partners, had engaged in unprotected anal intercourse with a casual partner, had had sex with a man met online, or had injected drugs in the 6 months prior to survey. One fifth (20.9%) of HIV infected men were undiagnosed; 1.3% of the total sample. Although HIV prevalence did not differ by ethnicity, HIV infected non-European respondents were more likely to be undiagnosed. Most of the small number of undiagnosed respondents had tested for HIV previously, and the majority believed themselves to be either "definitely" or "probably" uninfected. There was evidence of continuing risk practices among some of those with known HIV infection.

**Conclusions:**

This is the first estimate of actual and undiagnosed HIV infection among a community sample of gay men in New Zealand. While relatively low compared to other countries with mature epidemics, HIV prevalence was elevated in subgroups of MSM based on behaviour, and diagnosis rates varied by ethnicity. Prevention should focus on raising condom use and earlier diagnosis among those most at risk, and encouraging safe behaviour after diagnosis.

## Background

Men who have sex with men (MSM) are the group most implicated in HIV transmission in New Zealand, accounting for over three-quarters of all locally acquired infections diagnosed between 1996 and 2008 [1]. Since 2000, as in many developed countries, the annual number of new diagnoses in this group has increased [1]; in fact the largest number of MSM ever diagnosed in New Zealand was in 2010 [2]. Following the widespread introduction of effective antiretroviral treatment (ART) in 1996, life expectancy for people with HIV infection has improved [3,4]. Together with more new infections, reduced mortality has resulted in a doubling of the estimated number of MSM living with HIV in New Zealand between 1999 and 2009 [5]. This larger HIV prevalence pool would be expected to result in further increases in new infections unless there is a reduction in the annual transmission rate from infected MSM at a community level [3].

Early diagnosis can help reduce the infectivity of HIV positive individuals by motivating behaviour change and through timely antiretroviral treatment [6]. Those remaining undiagnosed, whether in the highly infectious early acute phase or prolonged latent phase of infection, play a disproportionate role in new HIV transmissions [7,8]. Mathematical modelling has suggested that in the Australian context, an estimated 9% of HIV-infected MSM with undiagnosed HIV infection could account for approximately 31% of new annual transmissions [9].

While the prevalence of diagnosed HIV among MSM has been estimated from self report in behavioural surveillance in New Zealand [10] and routine surveillance of new diagnoses [5], the only previous studies that have measured the true prevalence of HIV in this group have been among attenders at sexual health clinics [11]. However, those findings may not be generaliseable to the broader population of MSM at risk of HIV.

The aim of this study is to provide for the first time measures of the prevalence of HIV infection and the proportion undiagnosed in a community sample of MSM in Auckland, New Zealand. We also believe this to be the first internationally to introduce oral fluid specimen collection to an existing programme of behavioural surveillance where most participants are recruited from a large community event, enabling comparable measures of HIV prevalence to be collected in the future.

## Methods

Recruitment for the most recent Gay Auckland Periodic Sex Survey (GAPSS), a behavioural survey that has been undertaken every 2 years between 2002 and 2008 [10], took place in February 2011 at a gay community fair day in New Zealand's largest city Auckland, and over the subsequent week in the city's three gay bars and four sex-on-site venues. Participation criteria were having had sex with another man in the previous 5 years and being aged 16 or over. Sex was defined as "any physical contact you felt was sexual". At the gay community fair day, recruiters worked in teams from two large tents and approached men as they walked past. Recruiters invited men who agreed to self-complete the GAPSS questionnaire to also provide an oral fluid specimen using a specially designed collection device (OraSure Technologies, Inc., Bethlehem, PA, USA). All the main gay bars and sex-on-site venues in Auckland were approached and agreed to participate. In these venues, trained recruiters invited all men present to participate in the survey and to provide a specimen. Recruitment was scheduled over 3-4 h periods at different times of the week to generate a large and diverse sample. Both the questionnaire and oral fluid sample were anonymous, with the two linked by a non-identifying code. An information sheet was provided to all participants that made it clear they would not receive their individual test result, and provided details of HIV testing services in Auckland. All participants were offered a lollipop as a token of appreciation. Prior to fieldwork we undertook consultation with a number of stakeholders including indigenous Maori. The study received ethics approval from the Northern × Regional Ethics Committee of the New Zealand Ministry of Health. Details of the recruitment phase are described in Additional file 1.

The questionnaire contained the core items used in previous GAPSS rounds covering sexual partnering, sexual behaviours and condom use, attitudes, knowledge, HIV testing and demographic variables [10]. Those who had ever been tested for HIV were asked the result of their most recent test; and all, including those who had never been tested, were asked what they believed their present HIV status to be. In 2011, new items were added on injecting drug use and ART use.

Oral fluid samples were tested by the National Reference Laboratory (NRL) in Melbourne, Australia using an in-house version of the bioMériuex Vironostika Oral Fluid GACELISA test kit that had been developed for a previous study [12]. Specimens were initially tested for total saliva immunoglobulin G (IgG). Samples with adequate IgG were screened for HIV antibodies with the GACELISA. Positive samples were rescreened and if repeatedly reactive underwent confirmatory Western blot testing. The sensitivity and specificity of this process is believed to be 100% (95%CI: 95.0-100.0) and 100% (95%CI: 95.0-100.0) respectively [12].

The investigation of variations in HIV prevalence was informed by hypotheses that infection status would vary by patterns in exposure, by the underlying HIV prevalence among sexual contacts and by health seeking behaviours. Statistical analysis was performed using Stata version 11 [StataCorp, College Station, TX, USA). Pearson's chi-squared and Fisher's exact tests examined bivariate associations, and logistic regression was used to generate adjusted odds ratios (AORs) controlling for recruitment site, age and ethnicity.

## Results

One thousand three hundred and eighteen questionnaires were returned from 3791 invitations to participate, an acceptance rate of 48.5% of men approached who were considered likely to be eligible. This was 49.7% at the fair day, 52.3% at the gay bars, and 40.7% at the sex-on-site venues. Fourteen questionnaires were subsequently excluded due to incomplete responses or ineligibility.

Recruiters obtained 1073 oral fluid specimens from survey respondents. Five either contained no oral fluid or had very low levels of IgG, resulting in 1068 testable samples. Of these, 67 were repeatedly reactive on GACELISA and had a positive Western blot, and four were repeatedly reactive on GACELISA but negative on Western blot, two of which were deemed positives as their laboratory optical density data were close to the cut-off and these men reported being HIV infected and on ART. One positive and two negative samples were unlabelled, and a further 14 negative samples either had no corresponding questionnaire or were from ineligible or discarded respondents. Hence there were 1049 linked samples and questionnaires, of which 68 were from men deemed infected with HIV.

### Specimen provision rate

Collecting 1049 samples from 1304 eligible men resulted in an overall provision rate of oral fluid specimens of 80.4% enrolled in the survey. This varied by site, being highest at the fair day (82.3%), and lower at the bars (71.5%) and sex-on-site venues (76.5%) (Table 1). While there was no difference in specimen provision by any of the demographic characteristics, it did differ by certain aspects of sexual behaviour in the previous 6 months. It was lower among men with only one partner (76.6%), among those with more than 20 partners (73.3%) and among those with no recent casual partner (76.4%), and higher among those reporting unprotected anal intercourse with a casual partner (86.6%) and among those who had had a sexually transmitted infection (STI) in the previous 12 months (88.6%).

**Table 1:** Proportion of survey respondents who provided oral fluid specimens by recruitment site, demographic characteristics, sexual and other HIV risk and health seeking behaviour

	Number eligible	Specimen provided		p-value
		n	%	
**Recruitment site**				**0.006**

Fair day	994	818	82.3	

Gay bars	123	88	71.5	

Sex-on-site venues	187	143	76.5	

**Demographic characteristics**				

**Age**				0.52

16-29	454	367	80.8	

30-44	450	363	80.7	

45+	364	304	83.5	

**Ethnicity**				0.93

European	922	750	81.3	

Maori	136	111	81.6	

Pacific	37	29	78.4	

Asian	130	107	82.3	

Other	48	37	77.1	

**Highest education**				0.23

Less than tertiary degree	659	544	82.6	

Tertiary degree or higher	602	481	79.9	

**Sexual identity**				0.61

Gay	1122	908	80.9	

Bisexual	135	105	77.8	

Other	40	31	77.5	

**Residence**				0.43

Auckland	1023	834	81.5	

Other New Zealand	150	125	83.3	

Overseas	95	73	76.8	

**Sexual and HIV risk behaviour**				

**Sex with women < 6 months**				0.16

No	1196	969	81.0	

Yes	101	76	75.3	

**Number of male partners in previous 6 months**				**0.007**

0	68	57	83.8	

1	401	307	76.6	

2-5	438	371	84.7	

6-20	232	192	82.8	

> 20	116	85	73.3	

**Sex with casual partners in previous 6 months**				**0.007**

No casual partners	436	333	76.4	

Casual partners - no anal sex	189	158	83.6	

Casual partners - anal sex all protected	367	292	79.6	

Casual partners - anal sex not all protected	246	213	86.6	

**Sex with current regular partner in previous 6 months**				0.21

No current regular partner	542	445	82.1	

Regular partner - no anal sex	109	83	76.2	

Regular partner - anal sex all protected	193	148	76.7	

Regular partner - anal sex not all protected	391	325	83.1	

**Injecting drug use**				0.47

Never injected	1167	950	81.4	

Injected at least once in lifetime	90	76	84.4	

**Health seeking behaviour**				

**Sexual health check up in previous 12 months**				0.86

No	618	504	81.6	

Yes	658	534	81.2	

**STI in previous 12 months**			**0.05**	

No	1150	930	80.9	

Yes	105	93	88.6	

**HIV testing history**			0.17	

Last tested HIV negative	937	755	80.6	

Tested HIV positive	74	55	74.3	

Never tested	249	209	83.9	

**Total**	1304	1049	80.4	

Table omits data on respondents with missing information

### HIV prevalence

The overall prevalence of HIV infection was 6.5% (95% CI 5.1-8.1%). This was lower in those aged 16-29 years (3.3%), compared to those aged 30-44 (7.5%) and 45 years or older (8.9%) (Table 2). After adjusting for ethnicity and site of enrolment, the OR for HIV infection was significantly higher for those aged 30-44 years (2.4, 95% CI 1.2-4.9) and 45 years over (2.5, 95% CI 1.2-4.9), compared to men under 30. The prevalence was also higher among those resident overseas (13.7%) than those living in Auckland (6.0%) or elsewhere in New Zealand (4.8%); the AOR for overseas residents was significantly raised (2.2, 95% CI 1.1-4.8 adjusted for age, ethnicity and site of enrolment) compared to Auckland residents. There were no statistically significant differences by recruitment site, ethnicity, education or sexual identity.

**Table 2:** Prevalence of HIV infection and adjusted odds ratio by recruitment site, demographic characteristics, sexual and other HIV risk and health seeking behaviour

	HIV prevalence		AOR (95% CI)
	n/N	%	
**Recruitment site**			

Fair day	49/817	6.0	1

Gay bars	4/87	4.6	0.69 (0.24-2.0)

Sex-on-site venues	15/143	10.5	1.6 (0.85-3.2)

**Demographic characteristics**			

**Age group**			

16-29	12/366	3.3	1

30-44	27/362	7.5	**2.4 (1.2-4.9)**

45+	27/304	8.9	**2.5 (1.2-4.9)**

**Ethnicity**			

European	50/750	6.7	1

Maori	7/110	6.4	1.1 (0.47-2.5)

Pacific	3/29	10.3	2.0 (0.55-6.9)

Asian	3/107	2.8	0.41 (0.12-1.4)

Other	3/36	8.3	1.4 (0.41-4.8)

**Sexual identity**			

Gay	64/906	7.1	1

Bisexual or other	4/136	2.9	0.39 (0.13-1.1)

**Highest education**			

Less than tertiary degree	38/543	7.0	1

Tertiary degree or higher	26/480	5.4	0.76 (0.45-1.3)

**Residence**			

Auckland	50/832	6.0	1

Other New Zealand	6/125	4.8	0.78 (0.32-1.9)

Overseas	10/73	13.7	**2.2 (1.1-4.8)**

**Sexual and HIV risk behaviour**			

**Number of male partners in previous 6 months**			

0	2/57	3.5	0.54 (0.07-4.3)

1	11/307	3.6	1

2-5	22/369	6.0	1.6 (0.76-3.5)

6-20	19/192	9.9	**2.7 (1.2-5.8)**

> 20	11/85	12.9	**3.5 (1.4-8.7)**

**Casual male partnerships in previous 6 months**			

No casual partners	14/333	4.2	1

Casual partners - no anal sex	6/158	3.8	0.88 (0.32-2.4)

Casual partners - anal sex all protected	20/292	6.9	1.7 (0.81-3.6)

Casual partners - anal sex not all protected	25/211	11.9	**3.4 (1.7-6.9)**

**Regular male partnerships in previous 6 months**			

No current regular partner	26/445	5.8	1

Regular partner - no anal sex	3/83	3.6	0.62 (0.18-2.1)

Regular partner - anal sex all protected	12/148	8.1	1.5 (0.72-3.1)

Regular partner - anal sex not all protected	24/324	7.4	1.4 (0.75-2.5)

**Sex with a man met online**			

No	20/385	5.2	1

Yes but not in previous 6 months	18/339	5.3	1.2 (0.60-2.3)

Yes in previous 6 months	29/308	9.4	**2.3 (1.2-4.3)**

**Injecting drug use**			

No/not in previous 6 months	63/1008	6.3	1

Yes in previous 6 months	3/15	20.0	**4.9 (1.3-18.7)**

**Health seeking behaviour**			

**Sexually transmitted infection in the previous 12 months**			

No	60/928	6.5	1

Yes	6/93	6.5	0.93 (0.38-2.2)

**HIV testing**			

Tested at least once in lifetime	65/839	7.8	1

Never tested	3/202	1.5	**0.24 (0.07-0.79)**

Total	68/1047	6.5	

*AOR *adjusted odds ratio controlling for the effect of site, age and ethnicity *CI *confidence interval

Table omits data on respondents with missing information

There were a number of significant differences in HIV prevalence by recent risk behaviour after adjusting for recruitment site, age and ethnicity (Table 2). HIV prevalence rose with increasing numbers of sexual partners in the previous 6 months, being 3.5% and 3.6% respectively among those having none or one male sexual partner, 9.9% among those with 6-20 male partners (AOR 2.6, 95% CI 1.2-5.8 compared to one partner), and 12.9% among those with over 20 (AOR 3.5, 95% CI 1.7-6.9 compared to one partner in that period). Prevalence was higher among those who had any unprotected anal sex with a casual male partner in the previous 6 months (11.9%) (AOR 3.4, 95% CI 1.7-6.9 compared to those who did not have casual sex) and among respondents who had had sex with a male partner they had met online in the previous six months (9.4%) (AOR 2.3, 95% CI 1.3-4.3 compared to those who had never met a partner in this way). HIV prevalence was also significantly higher among the small number of respondents who had injected drugs in the previous six months (20.0%) (AOR 4.9, 95% CI 1.3-18.9). Conversely, HIV prevalence was lower among respondents who had never tested for HIV in their lifetime (1.5%) (AOR 0.24, 95% CI 0.07-0.79 compared to those who had tested at least once).

### Undiagnosed HIV infection

Of the 67 men with HIV infection who completed questions on past HIV testing and their most recent result, 53 (79.1%) had been diagnosed with HIV. Hence 14 of the infected men (20.9%, 95% CI 11.9-32.6) were unaware of their infection, a prevalence of unrecognised HIV of 1.3% (14/1046).

The prevalence of previously known and of unknown HIV infection, and the proportion diagnosed by demographic characteristics are described in Table 3. The small number (n = 14) of respondents with undiagnosed HIV infection precludes formal comparison of the proportion diagnosed by all of these characteristics. However, significantly more of those of European ethnicity (90%, 95% CI 78-97) had been diagnosed compared to all those of any other ethnicity when combined (50%, 95% CI 25-75) (Fisher's exact *p *= 0.002). This difference in diagnosis rates remained when limited to the 55 HIV positive respondents normally resident in New Zealand (90% among European versus 53% among non-European, Fisher's exact *p *= 0.005), a subset which is more relevant to informing HIV testing policy in this country.

**Table 3:** Prevalence of diagnosed and undiagnosed HIV infection, and proportion (95% confidence interval) of HIV infections diagnosed, by recruitment site and demographic characteristics

	Total	Diagnosed HIV	Undiagnosed	Proportion
		n (%)	HIV n (%)	diagnosed % (95% CI)
**Recruitment site**				

Fair day	816	39 (4.8)	9 (1.1)	81 (67-91)

Gay bar	87	4 (4.6)	0 (0.0)	100 (40-100)

Sex-on-site venue	143	10 (7.0)	5 (3.5)	67 (38-88)

**Age group**				

16-29	366	7 (1.9)	5 (1.4)	58 (28-85)

30-44	361	22 (6.1)	4 (1.1)	85 (65-96)

45+	304	23 (7.6)	4 (1.3)	85 (66-96)

**Ethnicity**				

European	749	44 (5.9)	5 (0.7)	90 (78-97)

Maori	110	4 (3.6)	3 (2.7)	57 (18-90)

Pacific Island	29	1 (3.5)	2 (6.9)	33 (1-91)

Asian	107	0 (0.0)	3 (2.8)	0 (0-71)

Other	36	3 (8.3)	0 (0.0)	100 (29-100)

**Sexual identity**				

Gay	905	51 (5.6)	12 (1.3)	81 (69-90)

Bisexual or other	136	2 (1.9)	2 (1.9)	50 (7-93)

**Highest education**				

Less than tertiary degree	542	34 (6.3)	3 (0.6)	92 (78-98)

Tertiary degree or higher	480	17 (3.5)	9 (1.9)	65 (44-83)

**Residence**				

Auckland	831	38 (4.6)	11 (1.3)	78 (63-88)

Other New Zealand	125	6 (4.8)	0 (0.0)	100 (54-100)

Overseas	73	8 (11.0)	2 (2.7)	0 (44-97)

**Total**	1046	53 (5.1)	14 (1.3)	79.1

*CI *confidence interval

Table omits data on respondents with missing information

### Characteristics of respondents without HIV and with known and unknown HIV infection

Tables 4 and 5 provide a description of respondents without HIV and with known and unknown HIV infection. Formal statistical comparisons are only made between the former two groups in view of the small number of men with unknown infection (n = 14).

**Table 4:** Behavioural characteristics of respondents without HIV infection, with known and with unknown HIV infection

	Uninfected	HIV infected	
		
	n (%)	Known n (%)	Unknown n (%)
**Number of male partners in previous 6 months**			

Up to 20	871 (92.2)	43 (82.7)*	10 (83.3)

> 20	74 (7.8)	9 (17.3)	2 (16.7)

**Casual male partnerships in previous 6 months**			

No casual partners	319 (34.3)	11 (21.2)†	3 (25.0)

Casual partners - no anal sex	152 (16.4)	6 (11.5)	0 (0)

Casual partners - anal sex all protected	272 (29.3)	13 (25.0)	6 (50.0)

Casual partners - anal sex not all protected	186 (20.0)	22 (42.3)	3 (25.0)

**Regular male partnerships in previous 6 months**			

No regular partner	419 (44.8)	22 (42.3)	4 (33.3)

Regular partner - no anal sex	80 (8.6)	2 (3.9)	1 (8.3)

Regular partner - anal sex all protected	136 (14.6)	9 (17.3)	2 (16.7)

Regular partner - anal sex not all protected	300 (32.1)	19 (36.5)	5 (41.7)

**Sex with a man met online**			

No	365 (37.8)	16 (30.8)‡	4 (28.6)

Yes but not < 6 months	321 (33.3)	13 (25.0)	4 (28.6)

Yes < 6 months	279 (28.9)	23 (44.2)	6 (42.9)

**Injecting drug use**			

No	897 (93.7)	38 (73.1)‡	12 (85.7)

Yes but not < 6 months	48 (5.0)	11 (21.2)	2 (14.3)

Yes < 6 months	12 (1.3)	3 (5.8)	0 (0)

**Sexually transmitted infection in previous 12 months**			

No	847 (91.2)	44 (89.8)	13 (92.9)

Yes	82 (8.8)	5 (10.2)	1 (7.1)

**Total**	979 (100.0)	53 (100.0)	14 (100.0)

* *p *< 0.05 for comparison between uninfected and known infected

† *p *< 0.001 for comparison between uninfected and known infected on dichotomised variable (last category versus rest)

‡ *p *< 0.05 for comparison between uninfected and known infected on dichotomised variable (first category versus rest)

Table omits data on respondents with missing information

**Table 5:** Responses to questions on attitudes to HIV-related issues of respondents without HIV infection, with known and with unknown HIV infection

	Uninfected	HIV infected	
		
	n (%)	Known n (%)	Unknown n (%)
**Condoms are OK as part of sex**			

Agree	935 (96.7)	48 (90.6)*	12 (100.0)

Disagree	32 (3.3)	5 (9.4)	0 (0)

**HIV/AIDS is a less serious threat than it used to be because of new treatments**			

Agree	296 (30.5)	28 (52.8)†	3 (23.1)

Disagree	675 (69.5)	25 (47.2)	10 (76.9)

**I would sometimes rather risk HIV transmission than use a condom during anal sex**			

Agree	135 (13.9)	7 (13.2)	0 (0)

Disagree	838 (86.1)	46 (86.8)	12 (100.0)

**I don't like wearing condoms because they reduce sensitivity**			

Agree	378 (39.1)	28 (52.8)	1 (8.3)

Disagree	588 (60.9)	25 (47.2)	11 (91.7)

**The sex I have is always as safe as I want it to be**			

Agree	886 (91.3)	46 (86.8)	10 (83.3)

Disagree	84 (8.7)	7 (13.2)	2 (16.7)

**Sometimes I feel under pressure to not use a condom**			

Agree	279 (28.8)	24 (45.3)‡	3 (25.0)

Disagree	690 (71.2)	29 (54.7)	9 (75.0)

**I'd like to be better informed about HIV transmission risk**			

Agree	734 (75.8)	35 (67.3)	10 (76.9)

Disagree	234 (24.2)	17 (32.7)	3 (23.1)

**I would never be willing to use condoms for anal sex**			

Agree	94 (9.7)	7 (13.2)	0 (0)

Disagree	875 (90.3)	46 (86.8)	12 (100.0)

**There is a "condom culture" among the men I have sex with**			

Agree	708 (75.7)	27 (54.9)†	7 (58.3)

Disagree	227 (24.3)	24 (47.1)	5 (41.7)

**A man who knows he has HIV would tell me he was positive before we had sex**			

Agree	371 (38.4)	10 (18.9)‡	5 (41.7)

Disagree	595 (61.6)	43 (81.1)	7 (58.3)

Total	979 (100.0)	53 (100.0)	14 (100.0)

Agree = "strongly agree" or "agree" with statement, Disagree = "strongly disagree" or "disagree" with statement

* *p *< 0.05 for comparison between uninfected and known infected

† *p *= 0.001 for comparison between uninfected and known infected

‡ *p *< 0.01 for comparison between uninfected and known infected

Table omits data on respondents with missing information

Table 4 shows the recent behaviour of the three respondent groups. Those with diagnosed HIV infection were significantly more likely than uninfected men to have had more than 20 partners in the past 6 months (17.3% versus 7.8%, *p *= 0.03), and to have had unprotected anal sex with casual partners (42.3% versus 20.0%, *p *< 0.001). Of the men with diagnosed HIV infection who had engaged in unprotected anal intercourse with casual partners, a similar proportion were on ART (64.8%) as those who did not have unprotected sex (60.7%) (*p *= 0.59). There were no differences in the proportion reporting a regular sexual partner nor the behaviour within this relationship between diagnosed positive and HIV negative respondents. However, men with known HIV infection who had any unprotected sex with a regular partner were much more likely to report that this partner was also HIV positive (68.4% versus 2.3%, *p *< 0.001). Men with diagnosed HIV were also more likely to have had sex with a man they had met on the Internet (44.2% versus 28.9%, *p *= 0.03), and to have injected drugs in the previous 6 months (5.8% versus 1.3%, *p *= 0.04). Similar proportions of men with known HIV infection (10.5%) and uninfected men (8.8%) had had an STI in the previous year.

Recent sexual behaviour of the men with undiagnosed HIV infection was not strikingly different from those with known infection (Table 4), but in view of the small number of the former statistical testing was not undertaken. Of these 14 respondents, 11 (78.6%) had previously tested for HIV; three in the last 6 months, five between 6 and 11 months prior, and three more than a year prior. Most (12/14, 85.7%) believed they were "definitely" or "probably" HIV negative at the time of survey, similar to the uninfected men (94.9%). One believed he was "definitely positive" and one stated that he "didn't know".

### Attitudes

Attitudes to HIV related issues are shown in Table 5 where again formal statistical comparisons are only made between the known infected and uninfected men. More of the former agreed that "HIV is less serious than it used to be" (52.8% versus 30.5%, *p *= 0.001). While the majority of men in both groups agreed that "condoms are OK as part of sex", this was reported by significantly more of the HIV negative men (96.7% versus 90.6%, *p *= 0.04). Uninfected men were more likely to agree that there was a "condom culture" among the men they have sex with (75.7% versus 54.9%, *p *= 0.001), and less likely to "sometimes feel under pressure to not use a condom" (28.8% versus 45.3%, *p *= 0.01). Furthermore, those with diagnosed HIV were less likely to agree that a man who knew he had HIV would disclose his HIV status before sex (18.9% versus 38.4%, *p *= 0.006).

## Discussion and conclusions

This is the first study to investigate HIV prevalence in a large, diverse community sample of gay men in New Zealand. Overall this was 6.5%, and higher among older men and those living outside New Zealand. Prevalence was markedly elevated among those with more sexual partners in the previous 6 months, those who had had unprotected anal sex with a casual partner, who had met a sexual partner online or who had injected drugs in that period. About one fifth (20.9%) of infected men were unaware of their infection. A lower proportion of ethnically European men were undiagnosed compared to other respondents. The vast majority of undiagnosed men thought they were HIV negative. Diagnosed HIV positive men were more likely to report more than 20 sexual partners, unprotected anal sex with casual partners, sex with a man they met online, and injecting drugs in the previous 6 months compared to HIV negative respondents. Uninfected men were more likely to exhibit attitudes conducive to HIV control.

We have demonstrated the acceptability of adding an anonymous oral fluid collection component into HIV behavioural surveillance undertaken at a large gay community event as well as gay bars and sex-on-site venues. The overall high specimen provision rate (80.4%) with little variation across enrolment sites, age and ethnicity suggest that the HIV prevalence estimates will be representative of those men taking part in the behavioural survey. As the survey is repeated in a consistent manner over time this will enable a comparable measure of HIV prevalence to be obtained in the future.

There are several limitations to the study. It is not possible to be certain of the actual response rate to the questionnaire as those who initially refused might have completed it subsequently, but it is probably in the order of half invited. The recruitment occurred in a number of community settings in Auckland so the findings may not be generalised to all gay men in New Zealand, to MSM in Auckland who do not attend these settings, or who only seek sexual partners through the Internet. Specimen provision was higher among men who had recently engaged in unprotected anal intercourse which may have resulted in slightly overestimating HIV prevalence among all study participants. While behavioural data rely on self-report that cannot be verified, the anonymity of the questionnaire and study protocols present little incentive to misreport. A small number of responses and biological data were inconsistent, with four respondents whose specimens were HIV negative on Western blot indicating they had tested HIV positive in their questionnaire. Two of the latter were deemed positive on the basis of them having relatively high optical densities on the original GACELISA test and reporting being on ART.

A prevalence of 6.5% in this Auckland study of MSM is consistent with past New Zealand clinic studies. In the most recent 2005/6 unlinked anonymous study of HIV among sexual health clinic attenders, the prevalence among MSM in Auckland was 6.1% [11]. Those who attend sexual health clinics would be expected to have a higher prevalence than those in a community sample as they have self identified as having STI risk. However, HIV prevalence in the latter is likely to have increased in recent years, with more infections having occurred coupled with prolonged survival.

Table 6 summarises the results from a number of prevalence studies among MSM in community settings internationally, with a wide range of findings. When placed alongside these, our data on HIV prevalence show that HIV remains relatively well controlled among Auckland MSM, despite having a mature epidemic that has existed since the early 1980s. This is consistent with the rate of new HIV diagnoses among MSM being lower in New Zealand than that in Australia, the United Kingdom and the USA [1]. As the rate of HIV diagnosis is higher in Auckland than elsewhere in New Zealand this conclusion will be valid for the whole country.

**Table 6:** HIV antibody prevalence and undiagnosed infection in community samples of MSM in selected countries

Location	Year conducted	Recruitment sites	Sample size*	HIV prevalence (%)	Proportion undiagnosed (%)	Ref
England (3 cities)	2003-4	GB, GS	348	8.6% Manchester	36.7% Manchester	[13,14]
				
			1436	12.3% London	44.1% London	
				
			373	13.7% Brighton	33.3% Brighton	

Scotland (2 cities)	2005	GB, GS	749	3.6% Glasgow	48.1% Glasgow	[14,15]
				
			601	5.5% Edinburgh	36.4% Edinburgh	

Southern Africa (3 countries)	2008	SB	537	17.4%	76.3%	[16]

United States (21 cities)	2008	GB, SO	8153	19.2% overall	43.5% overall	[17]
					
				6.4% Atlanta (low)	15.4% Seattle (low)	
					
				37.8% Baltimore (high)	73.4% Baltimore (high)	

Australia (2 cities)	2008	GB, GS	465	8.8% Brisbane	19.5% Brisbane	[18]

			639	9.5% Melbourne	31.1% Melbourne	[12]

France (Paris)	2009	GB, GS	886	17.7%	20%	

New Zealand (Auckland)	2011	GB, GS, CE	1049	**6.5%**	**20.9%**	[19]

*Number providing viable oral fluid sample

*GB *gay bars/club, *GS *gay saunas/sex-on-site venues, *SB *snowball sampling, *SO *social organisations, *CE *large community event

The proportion of HIV infected MSM who were undiagnosed (20.9%) in Auckland is considerably lower than found previously in Melbourne [12], five UK cities [13-15] and 21 US cities [17] (Table 6). This is somewhat surprising as behavioural surveillance shows that HIV testing rates among gay men in Auckland [10] are not higher than in those cities [14,17,20]. This may indicate that testing in Auckland is targeting MSM at highest risk, which since 2006 has included rapid HIV testing services [21], or the incidence is lower in Auckland.

Unlike the experience in other countries [22-24], we found no statistically significant differences in overall HIV prevalence by ethnicity in our sample. In particular, HIV prevalence was the same in European (6.7%) and in Māori (6.4%) MSM. This is consistent with previously reported epidemiological data suggesting no overrepresentation of HIV in this group, but it is different to the experience of indigenous individuals within MSM communities elsewhere [25]. We did on the other hand find that HIV positive MSM of non-European ethnicity were less likely to have had been diagnosed. This finding is consistent with a recent analysis that non-European MSM are more likely to present late in the course of infection in New Zealand [26] and warrants further investigation.

In spite of this favourable position internationally, the number of new diagnoses among MSM in New Zealand continues at a higher level than in the late 1990s [1]. Findings from this study add to our understanding of HIV transmission and acquisition risks, and have implications for ongoing prevention. We believe four issues should be highlighted.

First, we have shown high prevalence of HIV among MSM who had recently engaged in certain behaviours, including having over 20 recent sexual partners, having unprotected anal intercourse with casual partners, meeting sexual partners online, and injecting drugs. Men with these behaviours can act as ongoing reservoirs of infection especially if condoms are not used for anal sex, creating clusters of transmission if sexual contact is assortative (sexual mixing occurs with similar men), or dispersing HIV through sexual networks if contact patterns are dissortative. As most individuals will have imperfect information about the past behaviour of a prospective sexual partner, choosing to have unprotected anal intercourse may entail more risk than anticipated if this decision is based on an assumption that HIV prevalence is relatively low among all MSM.

Secondly, the majority of the men with undiagnosed HIV infection believed themselves to be HIV negative. Many had had a quite recent HIV test, and may consequently have held strong but incorrect convictions about their absence of HIV infection. These findings challenge safe sex strategies based on disclosure of HIV status alone. This is emphasised by our result that most of the diagnosed positive men would not expect a man who knew he had HIV to disclose this before sex; possibly a reflection of their own experience. Conversely the vast majority of all participants reported that condoms were "OK as part of sex", although the proportion disagreeing with this was higher (9.4%) among the known positive respondents. As condoms are a verifiable intervention by all participants during sex, they should continue to be strongly promoted.

Thirdly, the proportion undiagnosed varied among those with HIV. This was higher among men of non-European ethnicity, and also - although chance could not be excluded as the explanation for this finding - among those aged under 30 years. The reason for this needs to be explored further. Promotion of HIV testing should continue, and the responsiveness of testing services to diverse groups of MSM encouraged. All MSM with HIV infection should be able to access treatment as early as appropriate, which will not only be to their own benefit but also potentially reduce ongoing spread. More HIV testing, in terms of both coverage and frequency, should not however be considered the panacea for HIV control. The benefits of earlier diagnosis on ongoing transmission can only be expected if behaviour changes, and/or treatment that reduces infectivity is begun. HIV is particularly infectious in the weeks after infection, and modelling has shown that individuals in this primary stage of infection transmit disproportionately to their number in the community, but even very frequent routine testing would pick up few individuals at this stage. A negative HIV test should be seen as an opportunity to discuss HIV risk-taking, and how this could be reduced. To diagnose HIV infection after a number of negative tests should be seen as a failure to make full use of these opportunities.

Fourthly, recent behaviour that increases the probability of HIV transmission was more common among MSM with diagnosed HIV than among respondents who were uninfected. Risk of transmission exists, especially if individuals are not on ART [6,8], as was the case with 35.4% of diagnosed men in this study, or if HIV positive men have another STI [27], as was reported by 10% of our respondents in the previous year. We do not know if the behaviour of these individuals has changed since their HIV diagnosis, as would be suggested from previous overseas studies [28,29], but it does raise concern about ongoing spread by some diagnosed men in New Zealand. As ART was not reported universally among those with known HIV it is unlikely these men had all engaged in unprotected anal intercourse believing themselves to be uninfectious. Further research is needed to better understand these experiences. Overall, these findings suggest that the risk of onward transmission of HIV should continue to be emphasised by those providing care to MSM with HIV, and by their support communities.

## Competing interests

The authors declare that they have no competing interests.

## Authors' contributions

PS co-conceived the study, took overall lead on the project, analysed the GAPSS behavioural data and wrote the first draft. ND co-conceived the study and provided guidance on study design and interpretation. RG coordinated the oral fluid specimen collection. AH participated in study design and data collection. JR coordinated the GAPSS behavioural data collection. All authors read and approved the final manuscript.

## Pre-publication history

The pre-publication history for this paper can be accessed here:

http://www.biomedcentral.com/1471-2458/12/92/prepub

## Supplementary Material

Additional file 1 **Methods and fieldwork**. PDF file (text, table and figures), describes the planning, protocols and recruitment phase of the study. Click here for file
