# Supplementary material for: Actual and undiagnosed HIV prevalence in a community sample of men who have sex with men in Auckland, New Zealand
Source: BMC Public Health. 2012 Feb 1;12:92. doi: 10.1186/1471-2458-12-92 (PMC3293771; doi:10.1186/1471-2458-12-92)
Supplement: Additional file 1 — Methods and fieldwork. PDF file (text, table and figures), describes the planning, protocols and recruitment phase of the study. [file 1471-2458-12-92-S1.PDF]

Estimating actual HIV prevalence and undiagnosed infection in a community sample of men who have sex with men: Methods and fieldwork in a New Zealand oral fluid study

Richard Griffiths[1§], Peter JW Saxton[1*§], Nigel P Dickson[1], Anthony J Hughes[2], John Rowden[2]

[1]AIDS Epidemiology Group, Department of Preventive and Social Medicine, University of Otago Medical School, PO Box 913, Dunedin, New Zealand
[2]Research Unit, New Zealand AIDS Foundation, PO Box 6663, Wellesley Street, Auckland, New Zealand

*Corresponding author, email peter.saxton@otago.ac.nz
[§]These authors contributed equally to writing this fieldwork report
November 2011

Introduction
This report describes the methods of the first HIV prevalence study to be conducted among a community sample of men who have sex with men (MSM) in New Zealand. It highlights novel features of the project such as collecting oral fluid with indigenous MSM (Maori) and specimen collection at a large community fair day. It is intended to accompany the main summary paper [1] and to be read in conjunction with previous reports on the HIV behavioural surveillance programme in New Zealand (the Gay Auckland Periodic Sex Survey, or GAPSS) [2].

Background
MSM are the group most affected by HIV transmission in New Zealand, and the annual number of HIV diagnoses among this group increased during the early 2000s [3]. While routine surveillance of new HIV diagnoses provides information on known cases [4], less is understood about the true prevalence of HIV infection, which includes both known and undiagnosed infections. Previous studies have investigated actual HIV prevalence among MSM attending sexual health clinics in New Zealand [5], however this group may not be representative of the wider population of MSM at risk of HIV. Furthermore, the behavioural information collected in these studies has been limited, restricting the estimation of HIV prevalence to certain subgroups of MSM.

In October 2009, researchers from the AIDS Epidemiology Group, Department of Preventive and Social Medicine, University of Otago (AEG) and the New Zealand AIDS Foundation (NZAF) applied for funding from the Health Research Council of New Zealand (HRC) to investigate HIV prevalence among a community (i.e. non-clinic) sample of MSM. The aims of this study were to:

- estimate the prevalence of overall and undiagnosed HIV infection in a community sample of MSM in Auckland;
- identify the demographic, social network, and behavioural risk factors associated with undiagnosed infection;
- assess the practical feasibility (cost, protocols) and participant acceptability (specimen provision rate) of oral fluid specimen collection among this population.

The project subsequently received funding from the HRC (HRC# 10-418) in July 2010. It was also supported by the award of a University of Otago Health Sciences postdoctoral fellowship to Dr Peter Saxton, and funding from the Ministry of Health and the New Zealand AIDS Foundation.

The research team consisted of Dr Peter Saxton (AEG and principal investigator); A/Prof Nigel Dickson (AEG and principal investigator); Dr Richard Griffiths (AEG); Tony Hughes (NZAF); Dr John Rowden (NZAF).

Choice of method
Determining the true prevalence of HIV antibodies among a population requires the collection of biological as well as behavioural data. A number of studies estimating actual HIV infection among MSM have now been conducted internationally [6-21]. The majority have been one-off cross-sectional surveys where men were approached in clinical settings such as sexual health centres, or in community settings such as gay bars, and to a lesser extent gay saunas and cruise clubs (“sex-on-site” venues). The epidemiological context has also varied between locations with mature HIV epidemics (e.g. United Kingdom) to those with emerging HIV epidemics among MSM (e.g. China). These study populations both focus and limit the generalisation of findings to certain parts of the MSM population who are of particular public health interest. They also reflect the general challenges of obtaining more representative samples of gay and bisexual men that are large enough for reliable statistical inference in social and behavioural research [2,22].

For the New Zealand study, we proposed to introduce an oral fluid specimen collection as a component of the Gay Auckland Periodic Sex Survey (GAPSS) HIV behavioural surveillance programme, which has been ongoing since 2002. Factors influencing this decision included:

- GAPSS was already a collaboration between the research partners AEG and NZAF, with baseline funding from the Ministry of Health;
- GAPSS had established recruitment protocols that were held consistent over time. Using behavioural surveillance as a platform meant that HIV prevalence findings would be comparable if the study were to be repeated;
- GAPSS was well supported by the gay male community and media, and had engendered good relationships with community venues over time. This facilitated access to key data collection settings such as gay bars, sex-on-site venues and the annual Big Gay Out fair day;
- Previous experience meant there was a predictable sample size and diversity. Alternatively, the cost of obtaining a new sample with these characteristics specifically to estimate HIV prevalence would be prohibitive given likely funding options;
- The investigators believed that the GAPSS questionnaire was sound and would provide useful information on HIV prevalence among MSM subgroups with only minor additions to the instrument;
- Although recruitment would be limited to Auckland, this city has the largest population of MSM in New Zealand [22], with the highest prevalence of HIV based on routine surveillance [4] and clinic-based data [5].

Previously GAPSS had been conducted in February in 2002 (n=812), 2004 (n=1220), 2006 (n=1228), and 2008 (n=1527) [2]. The main amendment was a change in the GAPSS cycle from every two to every three years, with the next round scheduled for February 2011 due to preparation for the oral fluid component.

Oral fluid collection devices

The study used the Orasure® HIV-1 oral specimen collection device (OraSure Technologies, Inc., Bethlehem, PA) as this had been used in previous oral fluid studies conducted in similar settings. It was also preferred because our laboratory partner, the National Serology Reference Laboratory (NRL) in Melbourne, Australia had previously developed the capability for testing oral fluid specimens gathered by this device [17].

The Orasure kit consists of an absorbent pad attached to a plastic collection stick, and a specimen vial containing preservative fluid. The devices are easy to use which makes them suitable for non-health professionals working in the field. The device itself does not provide an HIV antibody test result and specimen testing occurs later at a laboratory. Thus they differ from devices such as the OraQuick rapid HIV test and other similar products.

The Orasure manufacturer's instructions detail best practice surrounding its use, handling, storage, transportation and laboratory testing procedures. Ideally, the device should be present in an individual's mouth for between 2-5 minutes, direct exposure to sunlight should be avoided, and the specimens should be stored between 2-37ºC and for no longer than 21 days, including the time required for transportation and laboratory testing.

The instructions also recommend that contamination via "foreign matter" should be avoided. Given that specimens were to be collected at a community fair day event and other social venues, we sought guidance from NRL regarding possible contamination if a participant was consuming alcohol, nicotine or other substances. Our laboratory partner indicated that while they had never experienced any problems associated with such substances, they cautioned against a participant putting the collection pad in their mouth if they had a mouthful of alcohol or a cigarette. These directions were incorporated into the training session for study recruitment staff.

Ethical issues

GAPSS survey programme

The GAPSS behavioural surveillance programme which provided the platform for the oral fluid study had been granted ethics approval at each successive survey round (Northern X Regional Ethics Committee 2001/301; AKX/03/12/336; NTX/05/12/164; NTX/05/12/164 PIS V#3 6/12/07). In brief, all men attending the recruitment settings aged 16 or over and who have had sex with a man in the previous five years are eligible to take part, regardless of whether they are usually resident in Auckland. Sex is defined as "any physical contact you felt was sexual". Participation is voluntary, anonymous, self-completed and study information sheets are provided with each questionnaire. Verbal agreement to take part implies consent. The questionnaire is typically 3 sides of A4 in length and takes most men between 5-12 minutes to complete. Completed questionnaires are placed by respondents directly into secure polling-style boxes and are not handled by recruitment staff.

Oral fluid collection

The additional collection of biological specimens for HIV antibody testing had to comply with New Zealand guidelines governing health research involving human tissue [23]. Similar research had been conducted in New Zealand among injecting drug users [24] and among sexual health clinic attendees three times previously [5]. The former collected anonymous oral fluid specimens, while the latter analysed anonymous unlinked blood samples

leftover from syphilis and/or hepatitis B serology. These studies had established that the collection of anonymous oral fluid and/or blood specimens for the purpose of epidemiological analysis was ethical in New Zealand under certain conditions (i.e. that participation is voluntary, anonymous, and that there is no potential for harm to participants).

Standard HIV antibody testing in New Zealand involves an ELISA screening test and a Western blot confirmatory test. In combination these provide 99.9% sensitivity and 100% specificity. Validation studies of oral fluid samples indicate sensitivity in the range of 99.7-100% and specificity between 99.0-99.8% [24]. The lower specificity is acceptable for research purposes in settings of medium HIV prevalence such as among MSM in New Zealand, since the positive predictive value would not be too adversely affected. Oral fluid testing is however not recommended for individual diagnostic purposes in New Zealand, meaning that results should not be communicated back to respondents. For this reason, as well as the anonymous nature of the study and the inappropriateness of delivering results to individuals in the recruitment setting (e.g. bars and public spaces), it was made clear to participants that they would not be able to receive their test results. Provision of an oral fluid specimen was optional for respondents completing a GAPSS questionnaire. Everyone was to be offered a card with contact details for testing services such as general practitioners, sexual health clinics and the NZAF Burnett Centre regardless of whether they had provided a specimen or not.

Cultural safety for indigenous Maori MSM was important for this study, as the collection and treatment of body parts is culturally sensitive. Prior to submitting the proposal to the Northern X Regional Ethics Committee, the investigators also sought advice from the Ngāi Tahu Maori Research Advisory Committee, a group affiliated with the Dunedin campus of the University of Otago (see “Consultation – Maori”).

The research team also consulted about the project with the NZAF Burnett Centre, which provides free and confidential rapid HIV testing and safe sex counselling in Auckland. Burnett Centre staff offered to reserve additional appointments during the recruitment week in case participation in the study raised concerns for some MSM.

Ethics approval for the GAPSS 2011 recruitment and oral fluid specimen collection was subsequently received from the Northern X Regional Ethics Committee in August 2010 (NTX/05/12/164 prot/amend 5/8/10).

Consultation

In the development phase of the study five stakeholder groups were identified: i) international research colleagues; ii) local gay bar and sex-on-site venue operators; iii) HIV community organisations; iv) Maori stakeholders; and v) Auckland MSM. Consultation was undertaken with all five groups.

International research colleagues

Following a review of the international literature, the research team contacted a number of other researchers who had conducted oral fluid studies among MSM in community settings. Engagement was sought with teams from Australia, England and South Africa as these were seen as being most applicable to our project in terms of study objectives and estimated HIV prevalence. Guidance was specifically requested over specimen storage and number code systems, pilot testing, selection of recruitment settings, marketing materials, fieldwork and participant recruitment (personal communication Joseph Debattista, Alisa Pedrana, Earl Burrell and Julie Dodds 2010). These exchanges enabled us to modify our methodological approach before engaging with local stakeholders. None of the research teams we contacted had collected oral fluid specimens at a large gay community fair event however, meaning that new protocols for this setting had to be developed.

Venue operators

The research team sent introductory letters to the owners of three gay bars and all four sex-on-site venues in the Auckland region. These described the aims of the oral fluid study and requested permission to have study recruitment staff on site. Meetings were held with each of the venue operators to answer questions, discuss recruitment details, possible shifts and placement of the research team in their venue. All venue operators approached agreed to be involved.

HIV community organisations

NZAF organises the annual gay pride community fair day held in February where GAPSS recruitment takes place. The research team liaised with NZAF staff organising this event to discuss how the oral fluid component would be incorporated into the 2011 recruitment round. As in previous years, permission was granted for GAPSS researchers to place two large tents in strategic positions at the park where the fair is held.

The researchers also consulted with Body Positive, an advocacy and peer support group for people living with HIV. As oral fluid collection was a new component to GAPSS, the research team felt that it was particularly important that men who knew they were HIV positive understood they were eligible to participate in 2011. Body

Positive offered useful insights on possible reactions from people living with diagnosed HIV, and offered to promote the aims of the study among its members.

Maori stakeholders
Consultation was also held with representatives from the indigenous Maori community. For many Maori, there are cultural protocols associated with the collection and handling of body tissue or fluids such as blood, urine, and saliva, as these fluids can be understood to hold a “tapu” or restricted state. Consequently body fluids should be kept separate from other materials such as food preparation areas and cooking utensils which are regarded as “noa” or neutral [26]. An important component of consultation was a “korero” or discussion about cultural protocols associated with the collection of oral fluid from Maori research participants.

Our research proposition was initially considered by the Ngāi Tahu Research Consultation Committee, a group which is affiliated with the University of Otago in Dunedin. While this group deemed the research to be of importance to Maori health, they recommended that consultation should also be held with Manawhenua or those who have the customary authority to speak on behalf of all Maori living in Tamaki Makaurau (the Auckland region) [letter of recommendation Ngāi Tahu Research Consultation Committee/Brunton 2009].

A focus group was convened in Auckland comprising of indigenous Maori MSM (or takatāpui – a term sometimes used by gay, lesbian, bisexual and transgender Maori to describe themselves as one who has “intimate companion[s] of the same sex”[27]). The research team sought feedback on study objectives, promotional materials, plans for the fieldwork, and study information sheets and cards. The group also pilot tested the Orasure collection device and discussed the taste and feel of the collection pad, potential barriers to participation and general comfort with taking part in the study. From this an agreement was reached on culturally appropriate ways of collecting oral fluid specimens from Maori respondents. For the fieldwork, this meant ensuring food or drinks were not placed on the tables where oral fluid specimens were being collected, nor stored in the portable coolers or refrigerators used to store the specimens. It was also emphasised that it would be inappropriate for recruiters to offer to insert or remove collection devices from the mouth, or to touch an individual’s head. Although specifically about cultural safety and appropriate engagement with Maori participants, it was evident that these practices would need to be applied to all participants regardless of their apparent ethnicity, as information on ethnicity would not be disclosed to recruiters at the time. Modifications to some of the recruitment protocols were made as a result.

As well as the takatāpui focus group, we approached Maori representatives of a local District Health Board where the modified protocols were discussed and deemed to be culturally appropriate for Maori participants.

Auckland MSM
A second focus group was also held with MSM actively involved in the Auckland community and recruited through community networks. Constituents were of diverse ages, ethnicities and HIV status. As with the Maori focus group, the research partners and aims of the study were presented, feedback was sought on promotional material, the oral fluid specimen collection devices were pilot tested and potential barriers to participation were discussed. Members completed an evaluation at the end of the session and the feedback was very positive.

Study promotion
The oral fluid component was a new addition to GAPSS and it was felt that a pre-study marketing campaign would be crucial for its success. A particular communication goal was avoiding potential confusion over whether the Orasure kit would provide an individual with their HIV antibody test result, either at the recruitment setting or later from a health provider. Feedback from the focus groups informed the promotional materials for the study. The study was branded “Get it Wet” and a variety of promotional mechanisms were used prior to and during the data collection. These comprised of:

- a dedicated website describing the oral fluid study, hosted by the University of Otago. This provided information about the study, how to provide a specimen, the research partners and FAQs (https://blogs.otago.ac.nz/oralfluidstudy);
- media releases introducing the study in electronic and print media (e.g. http://www.gaynz.com/articles/publish/2/article_9811.php);
- paid advertisements in electronic and print media;
- interviews on gay community radio shows;
- newsletters and websites of organisations such as NZAF;
- posters which were placed in gay bars and sex-on-site venues, and on the tents at the fair day;
- t-shirts worn by the research team featuring the slogans “Get it Wet” and “Oral Crew”;
- social media updates (e.g. Facebook) and word of mouth;
- support from gay community members and performers.

Training

A team of approximately 40 temporary, paid part-time staff was assembled to enrol men into the study. The majority of the team were male, and the recruiters represented a range of ages and diverse ethnic backgrounds including Pakeha/New Zealand European, Maori, Chinese, Indian, African and Middle Eastern ethnicities. The male bias was appropriate as the sex-on-site venues were male-only spaces. While a significant proportion of the recruiters openly identified as gay or bisexual, some male and female members of the team identified as heterosexual. Recruiters attended a three-hour training session before the fieldwork commenced. Topics covered the study aims, the sampling framework, the recruitment settings, the GAPSS questionnaire, study protocols and materials, confidentiality and recruiter and participant safety. All staff were provided with a recruiter manual.

Pilot testing

Role-play

The research team pilot tested the fieldwork protocols in two sessions. In the first session the research team and volunteers role-played the various steps from initial approach through to study explanation, questionnaire and oral fluid kit provision, specimen collection and handling, and survey deposit that were proposed for the fair day. Few guidelines surrounding oral fluid collection in this type of setting were apparent in the literature or through our consultation with colleagues overseas. This role-play proved invaluable as it revealed several limitations associated with our initial fieldwork design. For example, when respondent:recruiter ratios were high (i.e. at busy times of the event), it would be impractical for an individual to stand while filling out a questionnaire at the same time as unwrapping, administering and sealing their oral fluid specimen, have this timed accurately by a third party, return all completed material securely and be thanked in a manner than was consistent for all participants. As a consequence, an alternative system for organising data collection at the fair day event was developed. This led to the adoption of a Fordist-style production line process, involving division of labour and streamlined management of participants (see "Oral fluid specimen collection: Fair day event"). This was pilot tested a second time with the full recruitment team and further refinements were made.

Specimen Transportation

Several months before data collection we undertook a pilot test of specimen transportation processes between the research team in Auckland and the NRL laboratory in Melbourne, Australia to foresee possible delays, specimen deterioration or customs-related problems associated with official documentation and permits. The team contracted with Dangerous Goods International (DGI), a hazardous goods courier recommended by the NRL, to transport six known HIV negative samples by air. No problems were experienced during this trial. We also conducted a number of tests to ensure that the adhesive affixing the labels to specimen containers survived storage in various conditions, such as refrigeration and minor water damage. This was critical as the ability to link specimens to questionnaires hinged on the labels remaining in place. None of the labels suffered any notable damage.

Fieldwork

Selection of recruitment settings

GAPSS is designed as a repeated cross-sectional HIV behavioural surveillance among MSM attending social venues in Auckland [2]. While many health surveys use random national telephone sampling to generate participants, obtaining large numbers of MSM in this way is costly due to the low prevalence of homosexuality in the general population [28] and an absence of registers such as Census identifying precisely where homosexual men live [22]. Collecting repeat samples of ~1000 gay and bisexual men in this way is impractical given limited resources. In order to generate a large sample of MSM, the GAPSS project instead employs non-random techniques that target venues and events that attract large numbers of MSM, a technique that is described as "opportunistic" research [29-32].

When using non-random sampling in this way, behavioural surveillance must use methods that encourage participation amongst a wide variety of individuals if it is to generalise beyond an otherwise restricted group. For results to be comparable from period to period, recruitment strategies also need to be consistent each time so that biases between each of the study samples is minimised.

Since 2002 GAPSS site selection has been based around three types of settings: A gay community fair day event; gay bars; and sex-on-site venues. Together these have provided large samples of generally over 1200 respondents in the space of one week, and also diverse samples that include MSM of different ethnicities, age groups, socio-economic profiles, gay community affiliation and levels of sexual partnering. Due to the small number of venues in a city the size of Auckland, venue selection is not randomised as omitting any one of the existing venues would heavily bias that year's sample and make comparisons over time problematic. Instead, in 2011 as in previous years, all main venues were approached for their involvement following mapping and consultations and all agreed. The settings included in the 2011 recruitment were the Big Gay Out fair day event on 13 February, three gay bars and four sex-on-site venues (Table 1).

Over the one week data collection period in 2011, recruitment slots were scheduled for each venue using a similar logic employed for venue selection. In consultation with venue operators and with input from the NZAF, slots were chosen to maximise the sample and also to enable participation among different subsets of MSM who may be more likely to visit the venue at different times of the week. Some venues for example have student discounts or themed evenings on certain days of the week. Recruitment slots at the gay bars and sex-on-site venues were generally 3-4 hours long with 2-4 recruitment staff present. At the fair day event, recruitment occurred from midday until 6pm with 38 recruitment staff (Table 1).

Table 1 - Recruitment roster for GAPSS 13-20 February 2011

Venue	Sunday	Monday	Tuesday	Wednesday	Thursday	Friday	Saturday	Sunday
Fair day	12pm-6pm							
Gay bars								
Urge				9pm-1am	9pm-1am	10pm-2am	9pm-1am	
Family				8pm-12am	8pm-12am	9pm-1am	9pm-1am	
Lola						8pm-12am	8pm-12am	
Sex-on-site								
Centurian		3pm-7pm<br>7pm-11pm	3pm-7pm<br>7pm-11pm	3pm-7pm<br>7pm-11pm	3pm-7pm<br>7pm-11pm	10pm-2am	10pm-2am	1pm-5pm
Wingate		12pm-4pm		12pm-4pm		12pm-4pm	12pm-4pm	12pm-4pm<br>4pm-8pm
Lateshift		8pm-12am	8pm-12am	7.30pm-11.30pm	7.30pm-11.30pm	10pm-2am	10pm-2am	2pm-5pm
Basement			7.30pm-11.30pm		1pm-5pm	1pm-5pm		2pm-6pm

GAPSS respondent selection

GAPSS enrolment differs according to venue type. At the gay bars and sex-on-site venues, trained recruitment staff wearing study t-shirts and identification badges are instructed to approach all men present during their shifts who are accessible. At the fair day, recruiters work in teams from two large tents and approach men as they walk past. Participation in GAPSS is voluntary and individuals agreeing to take part are given a clipboard with a cover, which they can close over their questionnaire for privacy if they wish. The clipboards have a pen, a questionnaire and a study information sheet attached to them and respondents are instructed to complete the survey themselves. A respondent's questionnaire is anonymous as no names are requested or recorded. Verbal agreement to take part implies consent. Magnification sheets and glasses are provided at all venues for individuals with sight impairments. Respondents are instructed to place their completed surveys into one of the secure return boxes themselves and are not handled by recruitment staff in order to preserve the confidentiality of their responses. Response sheets are kept by recruitment staff recording whether an approach resulted in an acceptance, a refusal, whether the person had already completed the survey, or whether they were ineligible. Once an individual has submitted their completed questionnaire they are offered a bright yellow GAPSS-branded sticker signifying that they had taken part. This both promoted the study and avoided the individual being approached again by recruitment staff that day.

Oral fluid specimen collection

In 2011, men taking part in GAPSS were also invited to provide an oral fluid specimen. Those agreeing were given an Orasure specimen collection kit to administer themselves. A matching unique numbered sticker was simultaneously placed at the top of their questionnaire and on their specimen collection vial. Recruitment staff gave verbal instructions on how to provide a specimen and pictorial instructions were also available on laminated cards. Once the device was in their mouth, the individual was left to begin their questionnaire and timed using a three minute sand-timer. After three minutes the recruiter invited the participant to remove the device, place it in the collection vial and seal it. Recruiters temporarily stored specimen vials in portable coolers. On completion of their questionnaire the respondent was asked to remove it from the clipboard and place it into the secure return box. A recruiter thanked them, offered them a token of appreciation and a card with information about HIV testing services in Auckland.

Oral fluid specimen collection: Fair day event

Pilot testing established that the typical method of GAPSS recruitment at the fair day event had to be amended to accommodate practical issues surrounding oral fluid specimen collection. The solution devised was a Fordist production line system with division of labour and streamlined processes, illustrated in Figure 1.

This system responded to several potential problems at the busy community event. A focal entry point meant that roaming recruitment staff inviting men walking past to participate could direct or shuttle respondents to the front desk at one end of the tent where they were welcomed and handed clipboards. This maximised the time recruiters could promote the study and minimised the time spent explaining oral fluid processes and materials. At the front desk, GAPSS respondents were directed to sit down at a bank of trestle tables where they were invited by a recruiter seated opposite them to provide an oral fluid specimen while completing their questionnaire. As between 60-80 individuals were participating at the busiest time of the day, this system enabled recruitment staff to oversee several respondents concurrently. Information about the study and oral fluid instructions could then be delivered in an orderly way, recruiters did not lose track of how long the device had been in a person's mouth (an important element of specimen quality control), litter from the collection kits could be cleared quickly to centralised bins, and it provided a simple way for ensuring questionnaires and specimens were matched and completed specimens stored appropriately. Afterwards respondents were directed to the opposite end of the tent from where they entered and were offered tokens of appreciation (lollipops and sunscreen) and study information cards. The tents were large (6m x 12m), and the system allowed recruitment staff to coordinate their activities and reduced the likelihood that any important steps were overlooked.

Oral fluid specimen collection: Gay bars and sex-on-site venues
Lessons learned from the fair day event were brought into recruitment at the other venues. Each team was given a portable box of study materials and set up in a position agreed to by venue operators. In the sex-on-site venues this was always in the "dry" or "lounge" areas (as opposed to the wet sauna areas and private cubicles). As venues were often dark, reading lights were attached to all clipboards. Recruitment staff approached men in the same way as in previous GAPSS surveys and attempted to offer everyone accessible in the venue the opportunity to take part. If an individual agreed, they were invited to provide an oral fluid specimen as per the procedure at the fair day (Figure 2).

Completed specimens were stored temporarily in portable coolers which one member of the recruitment team supervised at all times. At the conclusion of the shift the completed questionnaires and specimens were collected by a member of the research team and returned to NZAF for secure storage. Oral fluid specimens were placed immediately in a refrigerator in a locked room.

GAPSS acceptance rate
GAPSS recruiters keep a record of invitations to take part in the survey so that response levels can be monitored. These indicate that at the gay community fair day, 992 approaches resulted in an accept, 1004 in a decline, and 565 in an "already completed" that day (an acceptance rate of 992/1996=49.7%). At the gay bars, records show 124 successful approaches, 113 declines, and 118 already completes (an acceptance rate of 124/237=52.3%). At the sex-on-site venues, records show 167 successful approaches, 243 declines, and 206 already completes (an acceptance rate of 167/410=40.7%). Overall the acceptance rate was 48.5% of approaches to eligible men.

Conventionally, response rates assume that the chance of contacting a sampled case is known and the likelihood of approaching the same person is extremely low. However this may not adequately capture willingness to participate in convenience-style surveys, where the possibility of repeat contacts may be high and where both participation and non-participation is anonymous.

For these reasons the "acceptance rate" recorded in GAPSS should be interpreted differently to the standard response rate recorded in other surveys, as it records the outcome of approaches by recruiters rather than the response of unique and eligible individuals. For example, by the Friday evening at the gay bar Family, the records show 23 accepts, 14 declines, 21 already completed previously that week, and 13 ineligible. It is possible that some of the 14 individuals who declined when approached will have agreed to participate later that evening, or later that weekend at the same or another recruitment venue. At the gay community fair day, recruiters also observed that some individuals often declined several times before agreeing to take part. This is supported by feedback from individual MSM who disclose an intention to participate that day, but when initially approached have been otherwise occupied (e.g. they were setting up a picnic, collecting lunch, browsing stalls with a group of friends etc). For example, an individual may have declined five times before agreeing to take part (=100% response rate), yet this is recorded as 5 different individuals declining and 1 accepting due to anonymity and multiple recruiters making approaches (=16.7% acceptance rate).

The research team believes this is an inescapable consequence of intensive recruitment drives in a small community using opportunistic sampling, where there is high circulation between venues. The sequence of recruitment at different venues over the one week data collection also needs to be considered, as do reports from recruiters that a point of saturation appears to be reached as the end of the recruitment week draws near.

Figure 1 – Recruitment at the community fair day event

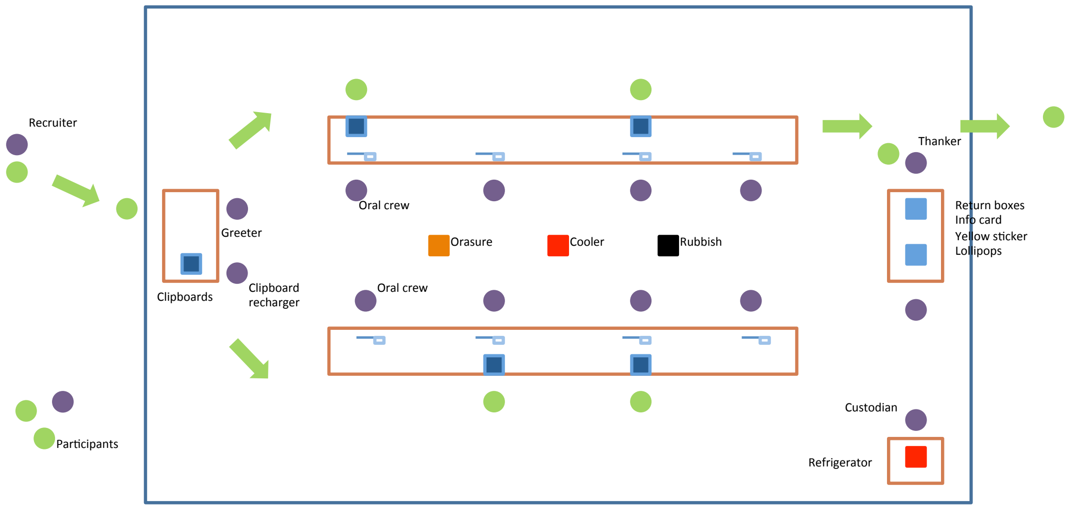

Figure 2 – Recruitment at the gay bars and sex-on-site venues

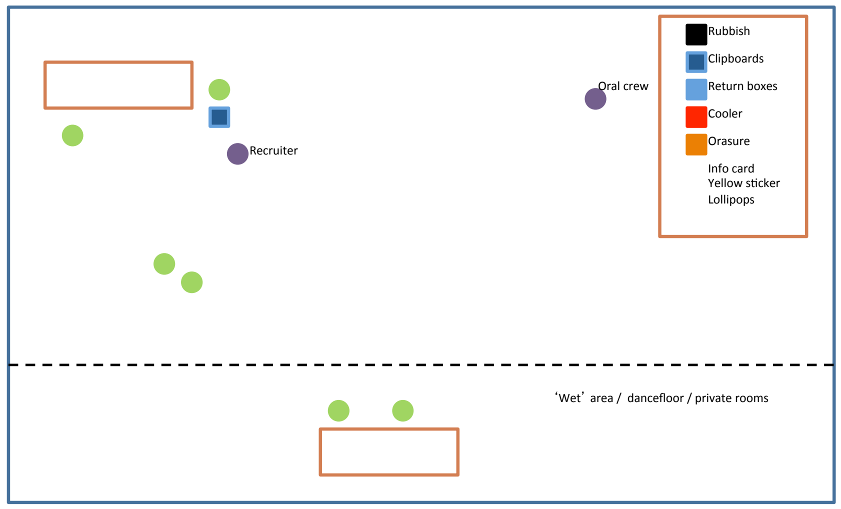

Linking specimens and questionnaires

Questionnaire coding

At the conclusion of the one week recruitment we received 1318 completed GAPSS questionnaires. These were entered into an electronic file by an independent data entry company. During data cleaning by the research team, 14 were deemed ineligible due to missing information on the majority of items, or the individual did not fulfil the study's eligibility criteria (for example they had not had sex with a man in the previous five years). Of the remaining 1304 eligible questionnaires, two were affixed with oral fluid specimen labels that could not be later matched to a corresponding label on an oral fluid specimen vial that had been analysed by the laboratory.

The majority of the GAPSS respondents were recruited from the community fair day (n=994, 76.2% of the total sample), with 123 (9.4%) recruited from the gay bars and 187 (14.3%) from the sex-on-site venues.

Laboratory analysis of oral fluid specimens

Oral fluid specimen containers were kept in a locked refrigerator at NZAF in Auckland during recruitment at approximately 4ºC. Each evening the collected specimens were placed in plastic zip-lock bags which were then dated. Before specimens were shipped to the NRL laboratory in Melbourne, Australia, members of the research team recorded the unique label code from each specimen into an excel spread sheet, which was provided to the laboratory at the conclusion of fieldwork.

We submitted 1073 oral fluid specimen containers to NRL for analysis in two batches, one after the community fair day and the second at the conclusion of recruitment in the gay bars and sex-on-site venues. Laboratory staff froze, processed and stored the specimens until they were scheduled for testing. Laboratory testing was conducted with an in-house version of the bioMériuex Vironostika Oral Fluid test kit that had been developed for a previous study [17].

Samples were initially tested for total saliva immunoglobulin G (IgG) to ensure that the sample was of adequate quality to enable NRL to detect HIV antibodies. Samples were then tested in the GACELISA to detect HIV antibodies. Any samples reactive in the GACELISA or close to the cut-off were retested in the GACELISA and then underwent confirmatory Western blot testing. A Western blot was considered positive if antibodies to the envelope gene were detected with or without the presence of antibodies to the other HIV specific bands. Only samples repeatedly reactive in the GACELISA and confirmed positive by Western blot were reported to the research team as positive.

The 1073 samples were tested for total IgG and on the GACELISA. A further 97 samples which were either reactive or close to the cut-off for the GACELISA were retested on the GACELISA and then underwent confirmatory Western blot analysis. The laboratory informed us that two specimen vials did not have a collection device inside. Of the remaining 1071, three were eliminated as their saliva IgG levels were too low to reliably detect HIV antibodies (Figure 3).

Of the 1068 viable samples, 67 were repeatedly reactive on GACELISA and Western blot and deemed positive by the laboratory. One thousand and one tested negative, four of which were repeatedly reactive on GACELISA but negative on Western blot. Two of these were subsequently deemed positives by the research team on further inspection of optical densities and self-reported responses (these two both stated that they had tested HIV positive, that they were currently on antiretroviral therapy, and that their current HIV status belief was "HIV positive"), leaving 69 positive and 999 negative samples. One HIV positive and two HIV negative samples were unlabelled from the fieldwork and could not be matched to a questionnaire (all were collected at the community fair day event). A further eight samples (all HIV negative) were labelled but no corresponding label was found on an eligible questionnaire, and eight samples (all HIV negative) were linked to one of the 14 GAPSS questionnaires deemed ineligible above. A flowchart summary of these results is provided in Figure 3.

Specimen provision rate

Ultimately 1304 eligible men completed the GAPSS questionnaire and 1060 viable specimens were collected from these men (1068 minus 8 who were deemed ineligible). Of these, 1049 could be matched to a questionnaire, an overall specimen provision rate of 80.4% (1049/1304).

Figure 3 – Oral fluid specimen flow chart

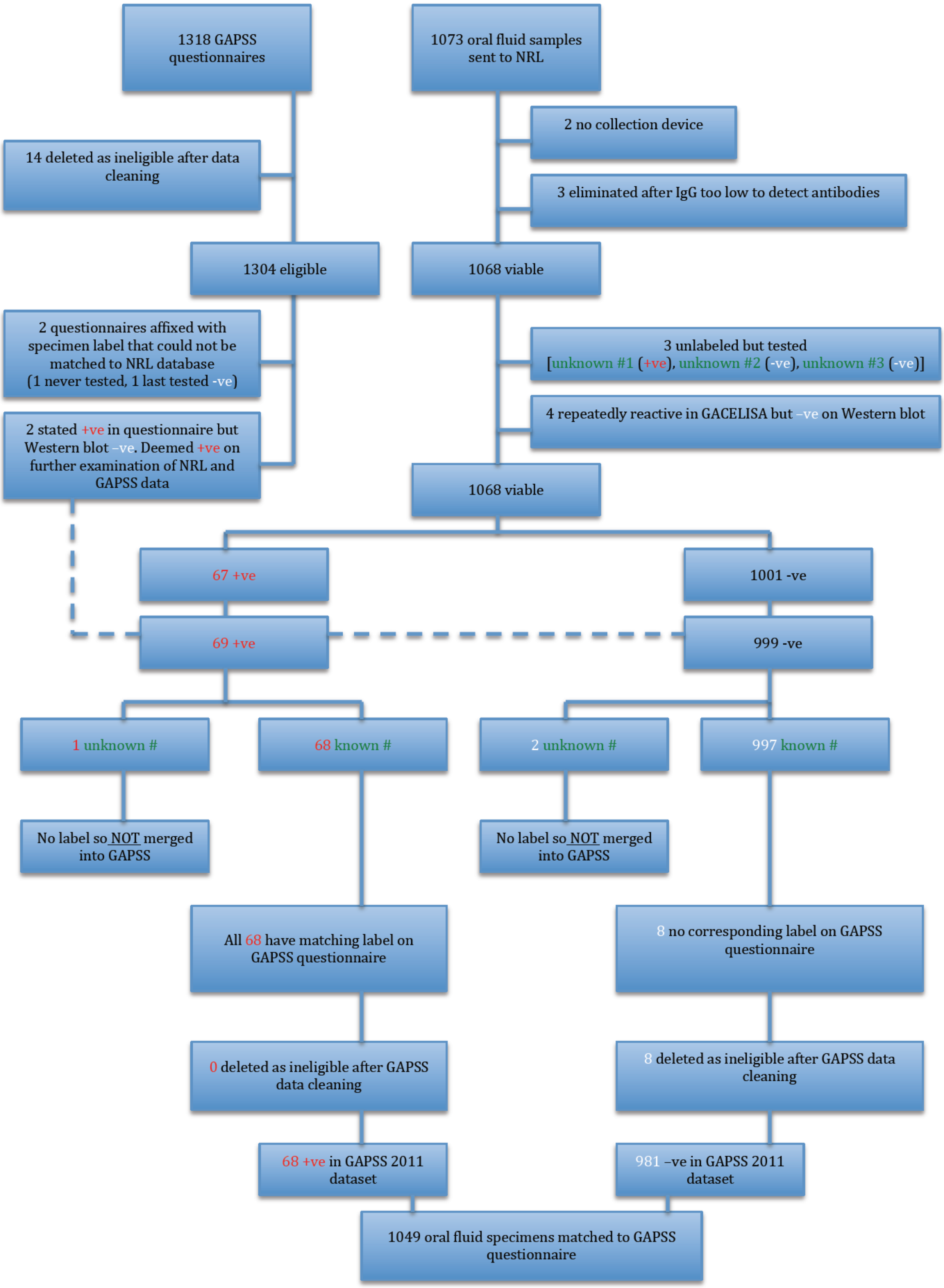

Conclusion

We have demonstrated the acceptability of collecting anonymous oral fluid samples to measure HIV prevalence in a large and diverse community sample of MSM in Auckland, New Zealand using an established behavioural surveillance programme as a platform. The linking of biological specimens with behavioural data from the surveys will enable the prevalence of undiagnosed HIV infection to be estimated, as well as whether HIV prevalence varies between subgroups of participants who may be of epidemiological or public health interest. We believe this is the first time both biological and behavioural data have been collected intensively in a large community fair day setting of MSM internationally, and the fieldwork protocols developed for this study may be of interest to others.

Acknowledgements

The authors would like to thank the Health Research Council of New Zealand, the Ministry of Health, the University of Otago Health Sciences Division and the New Zealand AIDS Foundation who provided funding. We gratefully acknowledge the stakeholders that were consulted in the development phase including the venues, NZAF and 'Get it On!' Big Gay Out team, Body Positive, Ngāi Tahu Research Consultation Committee, Nga Kai Tataki 'MRRC', and international research colleagues Joseph Debattista, Alisa Pedrana, Earl Burrell, and Julie Dodds. We would also like to thank the National Serology Reference Laboratory (Melbourne, Australia) who performed testing of the specimens and Dangerous Goods International for providing transportation of the samples. Finally, we warmly thank our study participants, recruitment team and all the Auckland venues, their staff and the focus groups for their time and involvement.

References

1. Saxton PJW, Dickson NP, Griffiths R, Hughes AJ, Rowden J: **Actual and undiagnosed HIV prevalence in a community sample of men who have sex with men in Auckland, New Zealand.** *BMC Public Health*, in press.
2. Saxton P, Dickson N, Hughes A: *GAPSS 2008: Findings from the Gay Auckland Periodic Sex Survey*. Auckland: New Zealand AIDS Foundation; 2010.
3. Saxton PJ, Dickson NP, McAllister SM, Sharples K, Hughes AJ: **Increase in HIV diagnoses among men who have sex with men in New Zealand from a stable low period**. *Sex Health* 2011, **8**:311-318.
4. Saxton PJW, Dickson NP, McAllister SM, Hughes AJ, Sharples K: **HIV prevalence among men who have sex with men in New Zealand 1985-2009: 25 years of public health monitoring**. *Int J STD AIDS*, in press.
5. McAllister SM, Dickson NP, Sharples K, Reid MR, Morgan JM, MacDonald EJ, Coughlan E, Johnston TM, Tanner NA, Paul C: **Unlinked anonymous HIV prevalence among New Zealand sexual health clinic attenders: 2005-2006**. *Int J STD AIDS* 2008, **19**:752-757.
6. Likatavicius G, Klavs I, Devaux I, Alix J, Nardone A: **An increase in newly diagnosed HIV cases reported among men who have sex with men in Europe, 2000-6: implications for a European public health strategy**. *Sex Transm Infect* 2008, **84**:499-505.
7. Gao L, Zhang L, Jin Q: **Meta-analysis: prevalence of HIV infection and syphilis among MSM in China**. *Sex Transm Infect* 2009, **85**:354-358.
8. van Griensven F, Thanprasertsuk S, Jommaroeng R, Mansergh G, Naorat S, Jenkins RA, Ungchusak K, Phanuphak P, Tappero JW: **Evidence of a previously undocumented epidemic of HIV infection among men who have sex with men in Bangkok, Thailand**. *AIDS* 2005, **19**:521-526.
9. Dodds JP, Johnson AM, Parry JV, Mercey DE: **A tale of three cities: persisting high HIV prevalence, risk behaviour and undiagnosed infection in community samples of men who have sex with men**. *Sex Transm Infect* 2007, **83**:392-396.
10. Williamson LM, Dodds JP, Mercey DE, Hart GJ, Johnson AM: **Sexual risk behaviour and knowledge of HIV status among community samples of gay men in the UK**. *AIDS* 2008, **22**:1063-1070.
11. Williamson LM, Hart GJ: **HIV prevalence and undiagnosed infection among a community sample of gay men in Scotland**. *J Acquir Immune Defic Syndr* 2007, **45**:224-230.
12. Sheridan S, Phimphachanh C, Chanlivong N, Manivong S, Khamsyvolsvong S, Lattanavong P, Sisouk T, Toledo C, Scherzer M, Toole M *et al*: **HIV prevalence and risk behaviour among men who have sex with men in Vientiane Capital, Lao People's Democratic Republic, 2007**. *AIDS* 2009, **23**:409-414.
13. Baral S, Trapence G, Motimedi F, Umar E, Iipinge S, Dausab F, Beyrer C: **HIV prevalence, risks for HIV infection, and human rights among men who have sex with men (MSM) in Malawi, Namibia, and Botswana**. *PLoS One* 2009, **4**:e4997.
14. CDC: **Prevalence and awareness of HIV infection among men who have sex with men – 21 cities, United States, 2008**. *MMWR* 2010, **59**:1201-1207.
15. Burrell E, Mark D, Grant R, Wood R, Bekker LG: **Sexual risk behaviours and HIV-1 prevalence among urban men who have sex with men in Cape Town, South Africa**. *Sex Health* 2010, **7**:149-153.
16. Birrell F, Staunton S, Debattista J, Roudenko N, Rutkin W, Davis C: **Pilot of non-invasive (oral fluid) testing for HIV within a community setting**. *Sex Health* 2010, **7**:11-16.
17. Pedrana AE, Hellard ME, Wilson K, Guy R, Stoove M: **High rates of undiagnosed HIV infections in a community sample of gay men in Melbourne, Australia**. *J Acquir Immune Defic Syndr*, epub ahead of print.

18. Mirandola M, Folch Toda C, Krampac I, Nita I, Stanekova D, Stehlikova D, Toskin I, Gios L, Foschia JP, Breveglieri M *et al*: **HIV bio-behavioural survey among men who have sex with men in Barcelona, Bratislava, Bucharest, Ljubljana, Prague and Verona, 2008-2009**. *Euro Surveill* 2009, **14**(48).
19. Kanter J, Koh C, Razali K, Tai R, Izenberg J, Rajan L, Van Griensven F, Kamarulzaman A: **Risk behaviour and HIV prevalence among men who have sex with men in a multiethnic society: a venue-based study in Kuala Lumpur, Malaysia**. *Int J STD AIDS* 2011, **22**:30-37.
20. Barin F, Velter A, Boutssou A, Guinard J, Plantier JC, Vu SL, Pillonel J, Alexandre A, Semaille C: **High prevalence of HIV-1 infection and diversity of circulating strains among men who have sex with men recruited in commercial gay venues in Paris (France): PREVAGAY 2009 Survey**. 18th Conference on Retroviruses and Opportunistic Infections (CROI) 2011, Boston. Abstract#:W-139.
21. German D, Sifakis F, Maulsby C, Towe VL, Flynn CP, Latkin CA, Celentano DD, Hauck H, Holtgrave DR: **Persistently high prevalence and unrecognized HIV infection among men who have sex with men in Baltimore: the BESURE study**. *J Acquir Immune Defic Syndr* 2011, **57**:77-87.
22. Hughes A, Saxton P: **Geographic micro-clustering of homosexual men: implications for research and social policy**. *Soc Pol J New Zealand* 2006, 28:158-178.
23. New Zealand Health and Disability Ethics Committees: **Guidelines for the Completion of the National Application Form for Ethical Approval of a Research Project NAFG-2009-v1.3.** Wellington: Ministry of Health, 2009.
24. Dickson NP, Austin FJ, Paul C, Sharples KJ, Skegg DC: **HIV surveillance by testing saliva from injecting drug users: a national study in New Zealand**. *J Epidemiol Community Health* 1994, **48**:55-57.
25. Delaney KP, Branson BM, Uniyal A, Kerndt PR, Keenan PA, Jafa K, Gardner AD, Jamieson DJ, Bulterys M: **Performance of an oral fluid rapid HIV-1/2 test: experience from four CDC studies**. *AIDS* 2006, **20**:1655-1660.
26. Sporle A, Koea J: **Maori responsiveness in health and medical research: key issues for researchers (part 1)**. *N Z Med J* 2004, **117**:U997.
27. Hutchings J, Aspin C: *Sexuality and the Stories of Indigenous People*. New Zealand: Hula Publishers; 2007.
28. Dickson N, Paul C, Herbison P: **Same-sex attraction in a birth cohort: prevalence and persistence in early adulthood**. *Soc Sci Med* 2003, **56**:1607-1615.
29. Mills S, Saidel T, Bennett A, Rehle T, Hogle J, Brown T, Magnani R: **HIV risk behavioral surveillance: a methodology for monitoring behavioral trends**. *AIDS* 1998, **12 Suppl 2**:S37-46.
30. Amon J, Brown T, Hogle J, MacNeil J, Magnani R, Mills S, Pisani E, Rehle T, Saidel T, Sow C: Behavioral Surveillance Surveys: *Guidelines for repeated behavioral surveys in populations at risk of HIV*. Arlington: Family Health International; 2000.
31. Pisani E, Lazzari S, Walker N, Schwartlander B: **HIV surveillance: a global perspective**. *J Acquir Immune Defic Syndr* 2003, **32 Suppl 1**:S3-11.
32. Brown T: **Behavioral surveillance: current perspectives, and its role in catalyzing action**. *J Acquir Immune Defic Syndr* 2003, **32 Suppl 1**:S12-17.
